# Canonical Correlation Analysis of Infant's Size at Birth and Maternal Factors: A Study in Rural Northwest Bangladesh

**DOI:** 10.1371/journal.pone.0094243

**Published:** 2014-04-07

**Authors:** Alamgir Kabir, Rebecca D. Merrill, Abu Ahmed Shamim, Rolf D. W. Klemn, Alain B. Labrique, Parul Christian, Keith P. West, Mohammed Nasser

**Affiliations:** 1 Department of Statistics, University of Rajshahi, Rajshahi, Bangladesh; 2 International Centre for Diarrhoeal Disease Research, Bangladesh (icddr,b), Dhaka, Bangladesh; 3 Center for Human Nutrition, Department of International Health, Bloomberg School of Public Health, Johns Hopkins University, Baltimore, Maryland, United States of America; 4 The JiVitA Maternal and Child Health and Nutrition Research Project, Gaibandha, Bangladesh; University of Nevada School of Medicine, United States of America

## Abstract

This analysis was conducted to explore the association between 5 birth size measurements (weight, length and head, chest and mid-upper arm [MUAC] circumferences) as dependent variables and 10 maternal factors as independent variables using canonical correlation analysis (CCA). CCA considers simultaneously sets of dependent and independent variables and, thus, generates a substantially reduced type 1 error. Data were from women delivering a singleton live birth (n = 14506) while participating in a double-masked, cluster-randomized, placebo-controlled maternal vitamin A or **β**-carotene supplementation trial in rural Bangladesh. The first canonical correlation was 0.42 (*P*<0.001), demonstrating a moderate positive correlation mainly between the 5 birth size measurements and 5 maternal factors (preterm delivery, early pregnancy MUAC, infant sex, age and parity). A significant interaction between infant sex and preterm delivery on birth size was also revealed from the score plot. Thirteen percent of birth size variability was explained by the composite score of the maternal factors (Redundancy, R_Y/X_ = 0.131). Given an ability to accommodate numerous relationships and reduce complexities of multiple comparisons, CCA identified the 5 maternal variables able to predict birth size in this rural Bangladesh setting. CCA may offer an efficient, practical and inclusive approach to assessing the association between two sets of variables, addressing the innate complexity of interactions.

## Introduction

There is a growing interest among maternal and child health researchers in studying the relationship between birth size and maternal socio-demographic and health factors. It is universally recognized that birth size is a lagged indicator of fetal health and predictive of neonatal health and survival. Birth weight in particular is strongly associated with mortality and morbidity in infancy and early childhood [1], [2]. However, fetal growth is largely, but not solely, determined by the availability of nutrients from the mother before and during gestation, as well as placental capacity to supply these nutrients in sufficient quantities to the fetus [3], and birth size can reflect the intrauterine environment.

Maternal nutritional status largely depends on socio-economic factors. Women from a higher socioeconomic status have increased access to and consumption of nutritious foods during or prior to gestation and more antenatal care (ANC) visits and nutrition supplementation during gestation. Small birth size is more common in resource poor settings or among more disadvantaged populations [4], [5], [6].

Birth weight is often the exclusive birth size measure used to evaluate fetal growth. However, other measurements like length and head, chest, and arm circumferences may be important in predicting long-term health and development outcomes [7]. When exploring the health effects of different exposures, observational epidemiologic studies often deal with data that include both a set of exposure variables and a set of outcome variables. Routine statistical approaches such as multiple linear regression used to analyze the relationship between exposures and outcomes such as birth size are usually challenged by the potential issues of multiple testing and multicollinearity [8], [9]. In some literatures, authors made an effort of analyzing birth size and other maternal, social or environmental variables [7], [10], [11], [12], [13] used multiple linear regression for analysis despite its limitations. Since CCA assesses the correlation between two composite variables called canonical variate, one representing a set of the exposure variables and the other a set of outcome variables [8], [9], it may be a useful method to evaluate the effect of maternal factors on infant's size at birth.CCA is the most general case of general linear model [9], [14], [15], [16] and thus it can be used to conduct the univariate and multivariate analyses that CCA subsumes, including multiple regression as a special case [17]. CCA has several advantages for researchers which were described elsewhere [18], [19]. Thus CCA is technically able to analyze data involving multiple sets of variables and is theoretically consistent with that purpose [9]. Although CCA is used currently in many branches of research: social and behavioral research [8], bioinformatics [20], genetics [21], neural network [22], environmental research [23] etc, it is relatively uncommon in public health research and to our knowledge, CCA has not been applied to analyze the relationship between maternal factors and birth size. The aim of this research is to explore the relationship between birth size and maternal factors using CCA in a community based maternal and child health and nutrition research project. We also want to identify the influential variables in the relationship and the significant interactions between variables.

## Materials and Methods

### Study design and participants

The data reported in this analysis were collected during a field based double-masked, cluster randomized, placebo-controlled trial assessing the efficacy of maternal vitamin A or β-carotene supplementation on maternal and infant mortality through 6 months of age from January 2002 to July 2007. Details are available elsewhere [24], [25], [26]. In brief, this study was conducted in a contiguous ∼435 sq km area in rural northwestern Gaibandha and Rangpur Districts of Bangladesh, with a population of ∼650,000. Predefined household clusters consisting approximately 250 households called sector (n = 596) were randomized to receive study supplements. Married women of reproductive age were enumerated through a baseline census and a subsequent 5 weekly surveillance was carried out to include newly married women. A 5-weekly visit was conducted to assess menstrual history. When a woman reported having missed her menstrual period in the past 30 days, pregnancy was confirmed using human chorionic gonadotropin based on the spot urine test. Once a woman was ascertained her pregnancy, she was asked for consent to receive study supplementation and providing data. Throughout the enrollment period 59721 pregnant women consented and enrolled into the trial [27].

On enrollment into the trial, mothers were interviewed about household socioeconomic conditions, education, demographic characteristics, previous pregnancy history, frequencies of dietary intake and morbidity in the previous 7 days and measured for mid-upper arm circumference (MUAC) [26]. A Living Standard Index (LSI) was constructed using principal component analysis from household socio-economic variables and was used as the main socio-economic variable [27]. Mothers were visited, provided allocated supplements (vitamin A, β-carotene or placebo) and checked for pregnancy and vital status throughout pregnancy to 3 months post-partum, at which time another interview was completed to obtain further data on maternal diet and morbidity, ANC, events and care during labor and delivery, and conditions of the infant.

Birth anthropometry was collected on infants of consenting mothers who took part in a placebo-controlled newborn vitamin A supplementation trial that was nested into the latter half of the above maternal trial [24]. Live-born infants (n = 21,585) were visited for dosing by field staff as soon as possible after birth (median (Inter Quartile Range, IQR) hrs: 7 (2, 18)). Of this number, 16,290 infants (75%) were singletons who were subsequently visited and measured by trained one of 56 anthropometrists within 72 hours of birth (median (IQR) hrs: 18 (9, 36) and included in the present analysis.

Birth size measurements included weight, length, MUAC and head and chest circumferences. Birth weight was measured to the nearest 10 g using a Tanita BD-585 digital pediatric scale (Tanita Corporation, Tokyo, Japan). Length was measured to the nearest 0.1 cm using an affixed headboard and movable footplate that had been fashioned for use with the Tanita scale. Circumferential measurements were made to the nearest 0.1 cm with a Ross insertion tape (Abbott Laboratories, Columbus, OH). All measurements, except for weight, were measured in triplicate with the median taken as the accepted value, as previously described [28]. The cut-offs used to define a small infant are, weight (<2.5 kg), MUAC (<10 cm), head circumference (<33 cm) and chest circumference (<30.5 cm) [29]. Among the 16,290 infants on whom birth anthropometry was collected, 14,506 (89%) had complete data and were included in the CCA which does not allow missing values.

The maternal characteristics included in the present analysis are: age at enrollment, parity, early pregnancy mid upper arm circumference (MUAC, cm), education (yrs), LSI, number of ANC visits, and maternal trial supplementation (Vitamin A or β-carotene). Additional infant characteristics included preterm (<37 week of gestation) delivery status and sex.

### Ethics Statement

The overall Jivita study protocol was reviewed and approved by both the Bangladesh Medical Research Council (BMRC) and the Institutional Review Board (IRB) of Johns Hopkins Bloomberg School of Public Health, Baltimore, Maryland, USA. Documented consent was given by all participating women.

### Canonical Correlation Analysis (CCA)

CCA is a multivariate statistical model that facilitates the study of linear interrelationships between two sets of variables: one set of variables is referred to as independent and the other as dependent; a composite score is formed for each set. CCA develops a canonical function that maximizes the correlation between the two composite variables. Additionally, CCA develops as many functions as there are variables in the smaller variable set; each function is independent (orthogonal) from the others so that they represent different relationships among the sets of dependent and independent variables [32]. The loadings of the individual variables differ in each canonical function and represent variables' contributions to the specific relationship being investigated. Now the challenge is to choose how many of them should be interpreted, however, in most cases the first function is the most legitimate. Hair et.al. [30] suggested 3 criteria of choosing the important functions as they believed that the use of a single criterion such as the level of significance is too superficial. Because the composite scores are calculated for each set to maximize the correlation between them, they don't care how much variability they take in to account of each set. The 3 criteria are: (i) level of significance (ii) magnitude of the canonical correlation, and (iii) redundancy measure for the percentage of variance accounted for from the two data sets like multiple regression's R^2^ statistic. We interpreted the most widely used test for significance of each function, the F statistic [31]. No generally accepted guidelines have been established regarding suitable sizes for canonical correlations. The decision is usually based on the contribution of the findings to better understand the research problem studied. The redundancy index [32] is analogous perfectly to the R^2^ statistic in multiple regressions. According to Sherry and Hensen [8], any function that explains <10% of the remaining variance after that explained by a certain number of functions, even if it has significant correlation, the effect sizes of the other functions are considered less impressive. In this paper we applied the criterion of a correlation significance level of 5% and redundancy coefficient of >0.10 to choose the interpretable canonical functions. CCA can be used for both continuous and categorical data of either dependent and independent variables [30].

To determine the relative importance of each original variable in to each function three methods have been proposed (i) canonical weights (standardized coefficients), (ii) canonical loadings (structural correlations) and (iii) canonical cross-loadings. As the canonical weights, like regression weights, are vulnerable to multicollinearity, most of the literature suggest to use canonical loadings or crossing loadings [9], [23], [30]. We used both loadings and cross loadings, however, there is no established cut off. There is a rule of thumb if any variable loading is >|0.30| then it can be considered to be an important contributing variable in to the function [33]. The score plot, 1^st^ variate on the horizontal axis and the 2nd variate on the vertical axis, of composite score also helped to find natural variable groupings in to the data set [34].

Multiple linear regression was used to examine the relationship between birth size and maternal factors and to compare the performance of the model with important maternal variables derived from CCA and the model with all maternal variables. Five models, one for each infant's size variable, were fitted with (i) 10 maternal factors and (ii) with only that factors which had significant loadings (≥30) in the canonical correlation analysis.

To support our CCA findings we stratified our samples by prematurity status and infant sex and investigated their interaction on birth size. We used mean and 95% CI of the 5 anthropometric measurements for 4 strata (Term-Female, Term-Male, Preterm-Female and Preterm-Male). Multivariate Analysis of Variance (MANOVA) was used to investigate interaction effects on infant's size at birth. All analyses were performed using statistical software R version 2.14.1. We used the CCA and yacca R packages.

## Results

More than 50% of the infants were born small. That is they were born with weight <2.5 kg, MUAC<10 cm, head circumference<33 cm and chest circumference<30.5 cm. Twenty seven percent of infants were preterm. Half of the infants were male. Mean (SD) maternal age was 22.0 (5.9) years and MUAC was 23.0 (2.0) cm. Most of the women (74%) had not reported an ANC visit. Nearly half of the women (∼43%) were nulliparous and their mean (SD) parity was 1.2 (1.4). Half of the women were literate (52%) and their mean (SD) years of schooling was 3.8 (3.9) (Table 1).

**Table 1:** Descriptive statistics for birth size and maternal socio-demographic factors from rural North West Bangladesh in 2002-2007, n = 14506.

Variables	Mean (SD)	Median (IQR)
**Birth size, within 72 hours of birth**		
Weight, kg	2.44 (0.42)	2.44 (2.18, 2.71)
Length, cm	46.43 (2.41)	46.50 (45.10, 48.00)
MUAC, cm	9.31 (0.84)	9.30 (8.80, 9.90)
HC, cm	32.36 (1.63)	32.50 (31.40, 33.40)
CC, cm	30.40 (2.09)	30.50 (29.20, 31.70)
**Maternal Socio-Demographic Factors**		
Parity	1.18 (1.41)	1.00 (0.00, 2.00)
Age at enrollment, year	21.96 (5.88)	21.00 (17.00, 26.00)
Early pregnancy MUAC, cm	22.99 (1.97)	22.90 (21.60, 24.10)
LSI	0.08 (0.96)	−0.11 (−0.65, 0.67)
Years of education	3.84 (3.86)	3.00 (0.00, 7.00)
No. of ANC visit	0.52 (1.15)	0.00 (0.00, 1.00)
Vitamin A supplementation	0.34 (0.47)	0.00 (0.00, 1.00)
β-carotene supp supplementation	0.33 (0.47)	0.00 (0.00, 1.00)
Preterm delivery^1^	0.27 (0.44)	0.00 (0.00, 1.00)
Infant sex	0.51 (0.50)	1.00 (0.00, 1.00)

ANC: Antenatal Care; CC: Chest Circumference; HC: Head Circumference; MUAC: Mid-Upper Arm Circumference; LSI: Living Standard Index.

1 Any delivery occurred before 37 weeks of gestation.

Table 2 represents the Pearson's correlation coefficient between the maternal factors and infant's size at birth. All maternal variables except preterm delivery and vitamin A or β-carotene supplementation were positively correlated with infant size at birth. All the infant's anthropometric measurements were negatively correlated with preterm delivery (P<0.05 for all), however, there was no correlation with maternal vitamin A or β-carotene supplementation.

**Table 2:** Pair wise Pearson's correlation coefficient, r (p-value), between the indicators of birth size, measured ≤72 hrs of birth, and maternal socio-demographic factors from north west Bangladesh in 2002-2007, n = 14506.

	Birth size				
Maternal factors	Weight, kg	Length, cm	MUAC, cm	HC, cm	CC, cm
Parity	0.14*	0.12*	0.13*	0.10*	0.14*
Age at enrollment, year	0.15*	0.13*	0.13*	0.10*	0.15*
Early pregnancy MUAC, cm	0.17*	0.13*	0.16*	0.13*	0.15*
LSI	0.10*	0.09*	0.09*	0.09*	0.09*
Years education	0.04*	0.04*	0.05*	0.05*	0.04*
No. of ANC visit	0.10*	0.09*	0.09*	0.08*	0.08*
Vitamin A supplementation	0.01	0.01	0.00	0.01	0.00
β-carotene supplementation	−0.02	−0.01	−0.01	−0.01	−0.01
Preterm delivery	−0.27*	−0.28*	−0.23*	−0.27*	−0.28*
Infant sex (M = 1, F = 0)	0.10*	0.12*	0.02**	0.18*	0.06*

ANC: Antenatal Care; CC: Chest Circumference; HC: Head Circumference; MUAC: Mid-Upper Arm Circumference; LSI: Living Standard Index.

* P<0.001, **P<0.01.

The canonical correlation coefficients and the redundancy indices are presented in Table 3. The CCA is restricted to deriving 5 functions because the dependent set contained the minimum number of 5 variables. The correlations for each successive function were 0.42, 0.19, 0.08, 0.04 and 0.02. All correlations except for the last were statistically significant (*P*<0.05, F-test). However, the redundancy index for all functions except the first one was zero. Therefore, only the first function is noteworthy in the context of this study.

**Table 3:** Canonical correlation analysis of birth size, measured ≤72 hrs of birth, and maternal socio-demographic factors from North West Bangladesh in 2002-2007, n = 14506.

Canonical variates	Canonical Correlation	F-statistic	P-value	Redundancy Index, R_Y/X_
Variate-1	0.422	71.977	<0.0001	0.131
Variate-2	0.192	18.483	<0.0001	0.004
Variate-3	0.079	4.939	<0.0001	0.000
Variate-4	0.036	1.933	0.019	0.000
Variate-5	0.024	1.417	0.204	0.000

The loadings and cross loadings of the variables for the 1^st^ canonical function are presented in Table 4. Looking at the loadings of the variables for function 1 the most important predictor of birth size was preterm delivery (loading: −0.74) followed by maternal early pregnancy MUAC (loading: 0.37), infant's sex (loading: 0.35), maternal age (loading: 0.34) and parity (loading: 0.32). Loadings of the birth size indicators demonstrated that all the anthropometric measurements similarly contributed to the first canonical function. So, all the infant's anthropometric measurements were most strongly negatively correlated with preterm delivery, and positively associated with maternal early pregnancy MUAC, infant sex, age and parity, in that order.

**Table 4:** Canonical weights, loadings and cross-loadings for the 1^st^ composite scores of the indicators of birth size, measured ≤72 hrs of birth, and maternal factors from North West Bangladesh in 2002-2007.

Variables	Loadings	Cross loadings
**Independent variables**		
Parity	0.32	0.13
Age, year	0.34	0.14
Early pregnancy MUAC, cm	0.37	0.15
LSI	0.23	0.10
Years of education	0.12	0.05
No. of ANC visit	0.23	0.10
Preterm delivery	−0.74	−0.31
Vitamin A supplementation	0.03	0.01
β-carotene supplementation	−0.03	−0.01
Infant sex (M = 1, F = 0)	0.35	0.15
**Dependent variables**		
Weight, kg	0.91	0.38
Length, cm	0.88	0.37
MUAC, cm	0.72	0.31
Head circumference (HC), cm	0.89	0.37
Chest circumference (CC), cm	0.87	0.37

ANC: Antenatal Care; CC: Chest Circumference; HC: Head Circumference; MUAC: Mid-Upper Arm Circumference; LSI: Living Standard Index.

Regression coefficients are presented in Table 5. In the models with all 10 maternal factors, except vitamin A and β-carotene supplementation all other factors were significant predictors of infant size at birth. However, in all the models with 5 maternal factors selected through CCA, all 5 factors were significant predictors of infant size at birth. The differences between the coefficients of determination, R^2^ of the full models and the models with 5 variables varied from 0.01 to 0.02.

**Table 5:** Regression analysis of influence of maternal factors on birth size using canonical correlation analysis.

	Weight, kg		Length, cm		MUAC, cm		CC, cm		HC, cm	
Predictors	Model 1*	Model 2*	Model 1	Model 2	Model 1	Model 2	Model 1	Model 2	Model 1	Model 2
	β (P-value)	β (P-value)	β (P-value)	β (P-value)	β (P-value)	β (P-value)	β (P-value)	β (P-value)	β (P-value)	β (P-value)
Parity	0.14 (<0.001)	0.10 (<0.001)	0.11 (<0.001)	0.07 (<0.001)	0.14 (<0.001)	0.09 (<0.001)	0.11 (<0.001)	0.07 (<0.001)	0.14 (<0.001)	0.10 (<0.001)
Age	0.07 (<0.001)	0.08 (<0.001)	0.08 (<0.001)	0.09 (<0.001)	0.05 (<0.001)	0.07 (<0.001)	0.05 (<0.001)	0.06 (<0.001)	0.07 (<0.001)	0.08 (<0.001)
Early pregnancy MUAC	0.12 (<0.001)	0.15 (<0.001)	0.08 (<0.001)	0.10 (<0.001)	0.12 (<0.001)	0.14 (<0.001)	0.09 (<0.001)	0.11 (<0.001)	0.11 (<0.001)	0.13 (<0.001)
LSI	0.05 (<0.001)	-	0.05 (<0.001)	-	0.05 (<0.001)	-	0.04 (<0.001)	-	0.05 (<0.001)	-
Education	0.04 (<0.001)	-	0.04 (<0.001)	-	0.04 (<0.001)	-	0.04 (<0.001)	-	0.04 (<0.001)	-
No. of ANC visit	0.06 (<0.001)	-	0.05 (<0.001)	-	0.05 (<0.001)	-	0.05 (<0.001)	-	0.05 (<0.001)	-
Male	0.11 (<0.001)	0.11 (<0.001)	0.13 (<0.001)	0.13 (<0.001)	0.02 (<0.001)	0.02 (0.003)	0.19 (<0.001)	0.18 (<0.001)	0.07 (<0.001)	0.07 (<0.001)
Preterm	−0.27 (<0.001)	−0.28 (<0.001)	−0.28 (<0.001)	−0.29 (<0.001)	−0.22 (<0.001)	−0.23 (<0.001)	−0.27 (<0.001)	−0.28 (<0.001)	−0.28 (<0.001)	−0.29 (<0.001)
Vitamin A supp.	0.00 (0.722)	-	0.00 (0.747)	-	−0.01 (0.308)	-	0.01 (0.297)	-	−0.01 (0.288)	-
β-carotene supp.	−0.02 (0.043)	-	−0.01 (0.401)	-	−0.01 (0.103)	-	0.00 (0.707)	-	−0.02 (0.025)	-
**R^2^**	**0.15**	**0.14**	**0.14**	**0.13**	**0.11**	**0.09**	**0.14**	**0.13**	**0.14**	**0.13**

MUAC: Mid-Upper Arm Circumference; CC: Chest Circumference; HC: Head Circumference.

*Model 1 consists of all variables and model 2 consists of variables for whose canonical loadings ≥0.30.

**Figure 1:**
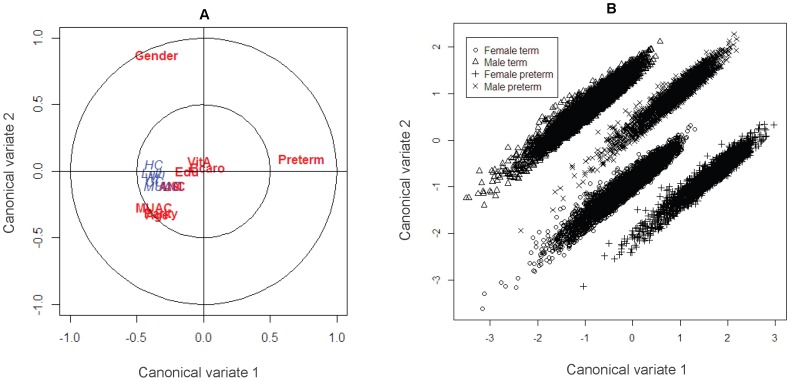
Biplot and score plot of the first two canonical functions. Panel **A** displays the biplot for the indicators of birth size and maternal factors with standardized weights indicated with blue and red, respectively. Panel **B** displays score plots for the maternal factors indicating any groupings among individuals.

Figure 1 shows the biplot of the standardized weights for the first two canonical functions for both the maternal factors and infant's anthropometric variables and score plot for the first two composite scores of the maternal factors. Panel **A** of Figure 1 illustrates that among the maternal factors preterm delivery had the greatest influence on first canonical function and infant's sex had greatest influence on the second canonical function but maternal early pregnancy MUAC, age and parity had similar influence on both functions and maternal vitamin A and β-carotene supplementation and maternal education had no influence on either function. The infant size variables had no influence on the second function which implies that most of the variability in infant size was accounted for by the first composite score. Panel **B** of the Figure 1 shows the score plot of the first and second composite scores of maternal factors. Four different groups among the infants are revealed. The grouping results from the interaction effect of preterm delivery and infant sex as they dominate the relationship.

Table 6 presents stratum wise mean and 95% confidence interval of birth size. Birth size was significantly different across stratum. MANOVA showed a significant interaction effect of preterm delivery and infant's sex on birth size; F = 161.83, p<0.001.

**Table 6:** Interaction effects of infant sex and preterm (gestation age <37 weeks) delivery on birth size, measured ≤72 hrs of birth from North West Bangladesh in 2002-2007.

	Term, Mean (95% CI)		Preterm, Mean (95% CI)	
Birth size	Female, n = 5300	Male, n = 5302	Female, n = 1822	Male, n = 2082
Weight, kg	2.46 (2.45, 2.47)	2.56 (2.55, 2.57)	2.21 (2.19, 2.23)	2.28 (2.27, 2.30)
Length, cm	46.53 (46.47, 46.58)	47.17 (47.11, 47.23)	45.02 (44.89, 45.14)	45.59 (45.47, 45.70)
MUAC, cm	9.41 (9.39, 9.43)	9.45 (9.43, 9.47)	8.97 (8.92, 9.01)	9.02 (8.98, 9.06)
CC, cm	30.61 (30.57, 30.66)	30.91 (30.86, 30.96)	29.27 (29.17, 29.38)	29.56 (29.46, 29.66)
HC, cm	32.31(32.27, 32.35)	32.95 (32.91, 32.99)	31.35 (31.27, 31.43)	31.88 (31.80, 31.96)
MANOVA: F = 161.83, p<0.001				

CC: Chest Circumference; HC: Head Circumference; MUAC: Mid-Upper Arm Circumference.

## Discussion

We studied the association between birth size and maternal factors using canonical CCA. CCA was used instead of separate linear regression models for each birth size measurement because it simultaneously models effects of multiple independent variables on multiple dependent variables. As CCA uses information from all the variables in both the exposure and outcome variable sets and maximizes the estimation of the relationship between the two sets, CCA may offer a more efficient approach for assessing the effects of the maternal factors on infant size at birth than methods routinely used, such as multiple linear regression. CCA starts with simultaneous consideration of both exposure and outcome variables, limiting the inefficiencies that may accompany conventional multiple testing, and, thus, reducing type-1 error. Furthermore, in CCA the latent variable approach, as used, helped to avoid multicollinearity [23]. The resulting procedure gives a global view of association between indicators of infant size at birth and maternal factors. We found that infant size at birth in rural Bangladesh had significant but moderate association with maternal nutritional and socioeconomic factors. In addition to providing an assessment of the association between two sets of variables, the application of CCA helped in narrowing down fewer exposure (maternal factors) and outcome variables (birth size) that might contribute to the relationship based on the variable loadings to the composite scores. Thus, CCA could be used as a comprehensive approach to extracting information from data to simultaneously identify both key exposure and outcome variables so that the assessment of the relationship between an individual exposure and an outcome can be further preceded. Additionally, CCA revealed a significant interaction between preterm delivery and infant's sex on birth size through the score plot of composite scores.

Because the birth size measurements are highly correlated, the combination of the indicators captures more information and, thus, as a composite variable may better predict future health outcomes more efficiently than use of a single birth size measure. For example, head circumference, as an indicator of brain volume [35], may provide important diagnostic and prognostic information, for example related to neurocognitive function [36], beyond that provided by birth weight alone. So too, might it be expected that, along with birth weight, other indicators of birth size like length and head, chest and arm circumferences can provide additional information about a wider range of health outcomes related to future child growth, health and development.

WHO suggests that a population with a prevalence of low birth weight of 15% or more or a prevalence of chest circumference at birth <30 cm experiences a disproportionately elevated risk of infant mortality and morbidity and long-term adverse effects on childhood growth and performance [37]. We found that approximately half of the infants in this typical rural, Bangladeshi population [25] were born both low birth weight [28] and small in chest circumference (<30 cm), revealing a major public health concern and a subset of infants whose health risks may extend beyond those associated with either criterion alone.

Pearson's correlation coefficients showed that maternal factors, age, parity, MUAC in early pregnancy, LSI of socioeconomic well being, maternal education, number of ANC visits and infant sex were significantly positively associated with birth size whereas, expectedly, preterm delivery was strongly negatively associated with newborn size measures. The individual multiple linear regression analyses also depicted virtually identical results, i.e. in all 5 models, except vitamin A and β-carotene supplementation, all other predictors had significant β-coefficients (*P*<0.05) (data not shown). Christian and colleagues [28] also found no significant effect of maternal vitamin A or β-carotene supplementation on newborn's anthropometry in the same population. CCA reduced the number of factors necessary to predict birth size to age, parity, early pregnancy MUAC, infant sex, and preterm delivery (loadings >0.30). If CCA was performed with these 5 predictors instead of 10 then canonical correlation would remain almost the same, ρ = 0.41 (data not shown). Thus, if CCA was not used before fitting the regression model we would have 3 redundant variables as significant predictors of infant's size. So in addition to evaluating the association between two sets of variables, CCA can also be used as a data mining tool in that it was able to narrow down fewer exposure and outcome variables which might contribute to the relationship.

The score plot of the composite scores can also identify the effect of interaction between factors on outcome of interest [34]. The composite scores are the projection of original multidimensional variables to a lower dimension subject to constraint that the correlation between the composite scores of dependent and independent variable sets is maximized. That is, the composite score for the maternal factors was constructed to mirror multiple dimensions of infant size at birth. The effect of interaction between independent variables on the dependent variables was depicted in the score plot of 1^st^ and 2^nd^ composite score of the independent variables. In this study, following the canonical correlation analysis, the multivariate analysis of variance indicated that infant sex and preterm delivery displayed a significant interaction effect on birth size. Infant size was bigger for the male term followed by female term, male preterm and female preterm. Many literature also found this kind of interaction effect on birth size [38].

In conclusion, CCA was used to explore the significant association between infant's size at birth and maternal factors. The maternal factors affecting or not affecting infant size at birth, isolated through canonical correlation analysis, were consistent with evidence of these kinds of associations in the literature [11], [12], [13], [39], [40], [41], [42], [43], [44]. CCA may offer an efficient, practical and more biologically comprehensive approach to assessing the association between two sets of variables, by taking into account the innate complexity of interactions and biological pathways that between variables.
